# The SMARTscreen Trial: a randomised controlled trial investigating the efficacy of a GP-endorsed narrative SMS to increase participation in the Australian National Bowel Cancer Screening Program

**DOI:** 10.1186/s13063-021-05877-3

**Published:** 2022-01-12

**Authors:** Anna Wood, Jon D. Emery, Mark Jenkins, Patty Chondros, Tina Campbell, Edweana Wenkart, Clare O’Reilly, Tony Cowie, Ian Dixon, Julie Toner, Hourieh Khalajzadeh, Javiera Martinez Gutierrez, Linda Govan, Gemma Buckle, Jennifer G. McIntosh

**Affiliations:** 1 Centre for Cancer Research, University of Melbourne, Melbourne, Australia; 2 Department of General Practice, University of Melbourne, Melbourne, Australia; 3 Melbourne School of Population and Global Health, Centre for Epidemiology and Biostatistics, University of Melbourne, Melbourne, Australia; 4 Healthily Pty Ltd, Melbourne, Australia; 5 Pen Computer Systems, Sydney, Australia; 6 Cancer Council Victoria, Melbourne, Australia; 7 Consumer representative. Healthily Pty Ltd, Melbourne, Australia; 8 Department of Software Systems & Cybersecurity, Monash University, Melbourne, Australia; 9 Department of Family Medicine, School of Medicine, Pontificia Universidad Católica de Chile, Región Metropolitana, Chile; 10 Western Victoria Primary Health Network Ltd, Ballarat, Australia

**Keywords:** Colorectal cancer screening, Bowel cancer, General practice, Health promotion, National Bowel Cancer Screening Program

## Abstract

**Background:**

Increasing participation in the Australian National Bowel Cancer Screening Program (NBCSP) is the most efficient and cost-effective way of reducing mortality associated with colorectal cancer by detecting and treating early-stage disease. Currently, only 44% of Australians aged 50–74 years complete the NBCSP. This efficacy trial aims to test whether this SMS intervention is an effective method for increasing participation in the NBCSP. Furthermore, a process evaluation will explore the barriers and facilitators to sending the SMS from general practice.

**Methods:**

We will recruit 20 general practices in the western region of Victoria, Australia to participate in a cluster randomised controlled trial. General practices will be randomly allocated with a 1:1 ratio to either a control or intervention group. Established general practice software will be used to identify patients aged 50 to 60 years old who are due to receive a NBCSP kit in the next month. The SMS intervention includes GP endorsement and links to narrative messages about the benefits of and instructions on how to complete the NBCSP kit. It will be sent from intervention general practices to eligible patients prior to receiving the NBCSP kit. We require 1400 eligible patients to provide 80% power with a two-sided 5% significance level to detect a 10% increase in CRC screening participation in the intervention group compared to the control group. Our primary outcome is the difference in the proportion of eligible patients who completed a faecal occult blood test (FOBT) between the intervention and control group for up to 12 months after the SMS was sent, as recorded in their electronic medical record (EMR). A process evaluation using interview data collected from general practice staff (GP, practice managers, nurses) and patients will explore the feasibility and acceptability of sending and receiving a SMS to prompt completing a NBCSP kit.

**Discussion:**

This efficacy trial will provide initial trial evidence of the utility of an SMS narrative intervention to increase participation in the NBCSP. The results will inform decisions about the need for and design of a larger, multi-state trial of this SMS intervention to determine its cost-effectiveness and future implementation.

**Trial registration:**

Australian New Zealand Clinical Trials Registry ACTRN12620001020976. Registered on 17 October 2020.

## Administrative information

Note: the numbers in curly brackets in this protocol refer to SPIRIT checklist item numbers. The order of the items has been modified to group similar items (see http://www.equator-network.org/reporting-guidelines/spirit-2013-statement-defining-standard-protocol-items-for-clinical-trials/).

**Table:** 

Title {1}	The SMARTscreen Trial: a randomised controlled trial investigating the efficacy of a GP-endorsed narrative SMS to increase participation in the Australian National Bowel Cancer Screening Program.
Trial registration {2a and 2b}.	Australian New Zealand Clinical Trials Registry (ANZCTR) ACTRN12620001020976 (http://www.anzctr.org.au/Trial/Registration/TrialReview.aspx?id=380259&isReview=true)
Protocol version {3}	Version 1.0 11th June 2021
Funding {4}	Victorian Cancer Agency project grant (CPRSRG19018)
Author details {5a}	Anna Wood^1,2^, Jon D Emery^1,2^, Mark Jenkins^3^, Patty Chondros^2^, Tina Campbell^4^, Edweana Wenkart^5^, Clare O'Reilly^6^, Tony Cowie^6^, Ian Dixon^7^, Julie Toner^7^, Hourieh Khalajzadeh^8^, Javiera Martinez Gutierrez^1,2,9^, Linda Govan^10^, Gemma Buckle^5^, Jennifer G McIntosh^1,8^ 1. Centre for Cancer Research, University of Melbourne, Melbourne, Australia 2. Department of General Practice, University of Melbourne, Melbourne, Australia 3. Melbourne School of Population and Global Health, Centre for Epidemiology and Biostatistics, University of Melbourne, Melbourne Australia 4. Healthily Pty Ltd, Melbourne, Australia 5. Pen Computer Systems, Sydney, Australia 6. Cancer Council Victoria, Melbourne Australia 7. Consumer representative 8. Department of Software Systems & Cybersecurity, Monash University, Melbourne, Australia 9. Western Victoria Primary Health Network Ltd, Ballarat, Australia 11. Department of Family Medicine. School of Medicine. Pontificia Universidad Católica de Chile
Name and contact information for the trial sponsor {5b}	The University of Melbourne is the trial sponsor. Phone: 13 MELB (13 6352) International: +(61 3) 9035 5511 Postal address: The University of Melbourne, Victoria 3010 Australia
Role of sponsor {5c}	The sponsor and funder do not have ultimate authority over the study design, management, data collection, analysis and interpretation of data, writing of the report and decision to submit the report for publication.

## Introduction

### Background and rationale {6a}

Australia and New Zealand have some of the highest rates of colorectal cancer (CRC) in the world [1]. In 2019, CRC was the fourth most commonly diagnosed cancer and the second commonest cause of cancer death in Australia. Currently, 40% of CRC is diagnosed at a late stage, leading to a poorer prognosis and outcome [2]. The simplest and most cost-effective method for reducing mortality from CRC is to detect precancerous or early disease through screening with a self-collected immunochemical faecal occult blood test (‘FOBT’) [3].

The Australian Government implemented a National Bowel Cancer Screening Program (NBCSP), sending people aged between 50 and 74 years a free home-based FOBT test kit every 2 years [4]. Despite the ease of access to the NBCSP testing kit, participation is low; only 44% of people return the completed kit. Women are more likely to complete the kit than men (46% compared with 41%) and older people are more likely to complete the kit than younger people (54% of 70 to 74 years old compared with 34% of 50 to 54 years old) [5]. Increasing participation in the NBCSP is a major Australian Government Health Department priority and has potential to significantly reduce morbidity and mortality associated with CRC as well as reduce unnecessary costs [6]. An increase in uptake of screening from 44 to 60%, will prevent 37,300 bowel cancer cases and 24,800 bowel cancer deaths and increasing participation in the NBCSP from 40% to 60% is estimated to reduce annual expenditure associated with CRC by a cumulative $AUD 1.7 billion up to 2030 and $AUD 2.1 billion between 2030 and 2040 [6].

The NBCSP involves primary care (‘general practice’) in the screening program only indirectly through the notification of tests and follow-up of positive results. There is strong evidence to suggest that GP endorsement of screening can increase participation in the NBCSP [7–9]. In Australia, four in five Australians have an episode of care with their GP annually [10], providing an opportunity to discuss CRC screening. To improve care and optimise GP time, general practices are increasingly using short messaging service (SMS) platforms linked to Electronic Medical Records (EMR), mainly to prompt and remind patients about appointments and routine health checks [11]. These messages, delivered directly to patients’ mobile phones, are considered a trustworthy source of health information by patients [12]. Communicating with the patients through SMS has many benefits. A message can be delivered in real time, can be accessed at the patient’s convenience on multiple occasions, and is sent to an individual’s personal device, so it is private and discreet [13]. SMS technology is increasingly being used regularly by older Australians with 82% of 50 to 60 years old accessing a smartphone daily [14]. Furthermore, the use of SMS to remind patients to complete the British Bowel Cancer Screening Program was found to be effective among patients aged 50 receiving a FOBT kit for first time [15].

Other methods that have demonstrated effectiveness at increasing the NBCSP uptake include using narrative communication or “storytelling” as a way of delivering a health promotion message. The Cancer Council of Victoria conducted a television advertising campaign using a highly relatable person talking positively about doing the NBCSP kit which resulted in an increased uptake of testing by 11%, with the highest impact among people who had never been screened or were not up to date with screening [16]. Another method demonstrated to increase screening by reducing apprehension about completing the test, especially self-collecting a faecal sample, has been to provide practical, easy-to-follow instructions about how to complete the NBCSP kit [17, 18].

These single interventions have had small effects on screening uptake [16, 19, 20]. This trial tests a combination of the four interventions within a single SMS (the ‘SMARTscreen intervention’) including (1) a GP endorsement of CRC screening, (2) video stories from relatable people who have had a positive experience about doing the test, (3) an advance notification video about how to complete the FOBT kit, and (4) information about the benefits of CRC screening and the NBCSP. The SMS is targeted to patients aged 50 to 60 years directly from their general practice as they are less likely to participate in screening, more likely to use smartphones, and evidence suggests once someone has done the NBCSP kit once, they are more likely to do it again [5]. The SMARTscreen trial aims to test the efficacy, feasibility, and acceptability of sending the SMARTscreen intervention as a prompt to patients prior to receiving their NBCSP kit on uptake of completing the kit.

### Objectives {7}

The primary objective is to test the efficacy of the SMARTscreen intervention on participation in the NBCSP up to 12 months in people aged 50 to 60 years who are due to receive an FOBT kit from the NBCSP compared to those who receive usual care.

The secondary objective is to determine the feasibility and acceptability of the SMARTscreen intervention from the general practice staff as well as the patients’ perspectives by conducting a process evaluation.

#### Hypotheses

The use of a narrative SMS delivered to patients aged between 50 and 60 years old and due for colorectal cancer screening from their general practice, just prior to receiving the NBCSP FOBT testing kit, will increase the likelihood of completing the kit compared to usual care.

### Trial design {8}

We will conduct a superiority cluster randomised controlled efficacy trial to test if patients in the general practices who receive the SMARTscreen intervention are more likely to complete the NBCSP kit compared with patients in the control group general practices. The unit of randomisation will be the general practice allocated in a 1:1 ratio to either the intervention or control group stratified by practice location and practice size.

Embedded in the trial will be a process evaluation using quantitative and qualitative methods. The quantitative measures will include the number of people who view each of the four individual intervention components of the SMARTscreen SMS and how often they view them. The qualitative research will explore the feasibility and acceptability of the intervention by general practice staff (including GPs, practice managers, nurses, other administrative staff) and patients. This will be done using semi-structured interviews with staff and patients about their experience of sending SMS intervention monthly to their patients (staff) or receiving the SMS (patients).

## Methods: Participants, interventions and outcomes

### Study setting {9}

The SMARTscreen trial will be conducted in general practices located in the Western Victorian Primary Health Network (WVPHN) [21] catchment in Victoria, Australia, which includes a mix of regional cities and towns, and socio-economic and demographic diversity. The population of the WVPHN catchment includes 618,000 people, of which 190,934 (30.4%) are between 50 and 74 years old, the age targeted by the NBCSP [22, 23]. The WVPHN is also a partner in this study.

### Eligibility criteria {10}

#### Inclusion criteria for general practices

General practices will be eligible to participate in the SMARTscreen trial if they are located in the WVPHN region, their electronic medical record (EMR) system is compatible with the Pen CS CAT4 clinical audit tool software (CAT: Pen Computer Systems; Leichardt, NSW, Australia) [24] and the SMS recall platform called Go Share Plus [25] developed by Healthily (an Australian-based health technology and patient education company). To ensure there will be enough eligible patients, each general practice will have to have at least two full-time equivalent GPs and all GPs will have to agree to consent to allowing their eligible patients to receive the SMS. CAT4 is linked to the EMR and is used to generate lists of patients and their mobile telephone numbers due to receive the NBSCP kit based on the eligibility criteria. The SMS will be sent to patients from the Go Share Plus platform.

#### Inclusion and exclusion criteria for patients

Inclusion criteria are active patients of the general practice aged between 50 and 60 years old and due to receive a NBCSP kit in the next month based on their date of birth or date of completion of the NSBCP kit. NBCSP kits are sent shortly after a person’s 50th birthday and then every 2 years. If a person completes a kit the date for the next kit is 2 years after the date of completion.

An active patient is defined as someone who has at least three episodes of care recorded at the general practice within the previous 2 years [26]. Only active patients are included so that we do not inadvertently send an SMS to patients who have another principal general practice where their NBCSP results are recorded.

Patients will be excluded from the trial if they do not have a mobile telephone number recorded in their EMR record, have opted out of receiving SMS messages from their general practice, or have a diagnosis of CRC recorded in the EMR.

### Who will take informed consent? {26a}

#### General practice informed consent for the trial

The trial coordinator will obtain informed consent from all eligible and interested general practices. The trial coordinator will discuss the trial with the practice staff at a meeting organised by the practice manager. This will be conducted by an electronic meeting system (EMS) (e.g. Zoom), due to the COVID-19 pandemic [27] and associated physical distancing restrictions. The practice manager will be sent the plain language statement and consent form via email following a telephone conversation to discuss SMARTscreen and gauge interest. A Plain Language Statement brochure (Additional Document A) will be sent to all staff in the general practice by Australia Post. The general practice manager and lead GP or their representative will complete the consent form on behalf of the general practice staff, and a signed copy will be sent back to the trial coordinator.

#### Patient informed consent for the trial

Patients will not provide individual consent as no identifying information about the patients will be collected or viewed by the research staff. Patients routinely receive SMS text messaging information and reminders from their general practice unless they individually seek to opt out of receiving information in this way or do not have a mobile number recorded in their EMR.

Patients can also remove themselves from the trial either by informing the practice manager or by using the ‘opt out’ instructions in the SMARTscreen SMS. Only aggregated, de-identified and non-re-identifiable patient data will be included in the study results.

The SMARTscreen trial will be advertised with posters in the waiting rooms of participating general practices to inform people that research is being undertaken in the general practice and they can request more information about this research.

#### General practice staff and patient informed consent for the qualitative interviews

Individual informed consent will be obtained from all interviewees. General practice staff working in intervention general practices who are involved in the delivery of the SMARTscreen intervention will be invited to participate in a semi-structured interview (Additional Document B). They will receive a plain language statement and consent form explaining the purpose of the interview. A trained qualitative interviewer who is not involved in the SMARTscreen trial will contact the general practice staff to obtain informed consent and conduct semi-structured interviews either by zoom or face-to-face depending on interviewees preference.

Intervention group practice managers will invite eligible patients to participate in an interview using a sampling matrix to ensure a range of patients are interviewed based on demographics: location (rural or regional city), age and sex. Patients who consent to be contacted by research staff will be given a Plain Language statement and Consent Form. The trial coordinator will contact consenting patients to obtain informed consent to participate in an interview (Additional Document C) at a time of their choice. All interviews are conducted by trained qualitative interviewers and all interviewees will be re-imbursed for their time with an AUD$50 gift voucher.

### Additional consent provisions for collection and use of participant data and biological specimens {26b}

Not applicable. No biological specimens will be collected.

## Interventions

### Explanation for the choice of comparators {6b}

General practices allocated to the control group will continue their usual care which will include any methods they currently use to promote participation in the NBCSP. Patients’ healthcare will not be compromised, and general practices will continue to support patients who test positive through the NBCSP as per their usual care practice.

### Intervention description {11a}

The SMARTscreen SMS will be sent to eligible patients from general practices in the intervention group in the last week of each month of the 6-month intervention phase. Staff use the CAT4 software, which is linked to the EMR to generate the list of eligible patients using the custom made SMARTscreen filters within CAT4. These filters were designed for the SMARTscreen trial and developed by Pen CS to identify lists of patients by age (patients who are due to turn 50, 52, 54, 56, 58, or 60 years old in the current month), have not had a diagnosis of CRC, and are eligible to receive the NBCSP kit in the next 4 weeks. An additional filter was developed to identify the group of patients aged between 50 and 60 years who have a FOBT result recorded 24 months prior and therefore due to receive a NBCSP kit. GoShare Plus is a recall SMS messaging platform integrated within CAT4 which sends the SMARTscreen SMS to the list of generated patients’ mobile telephone numbers.

#### The SMARTscreen SMS intervention

**Fig. 1:**
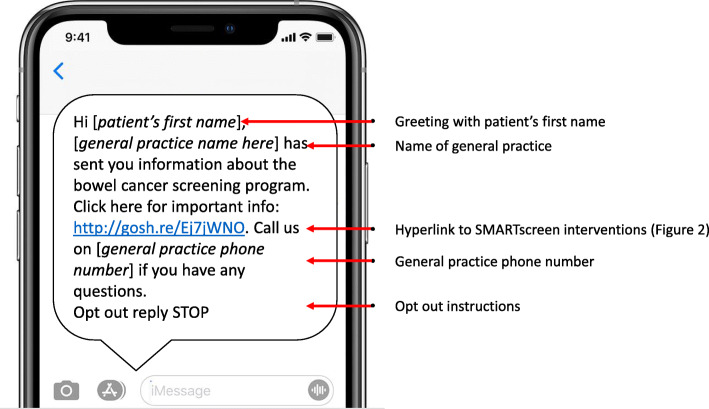
SMARTscreen SMS - test message

**Fig. 2:**
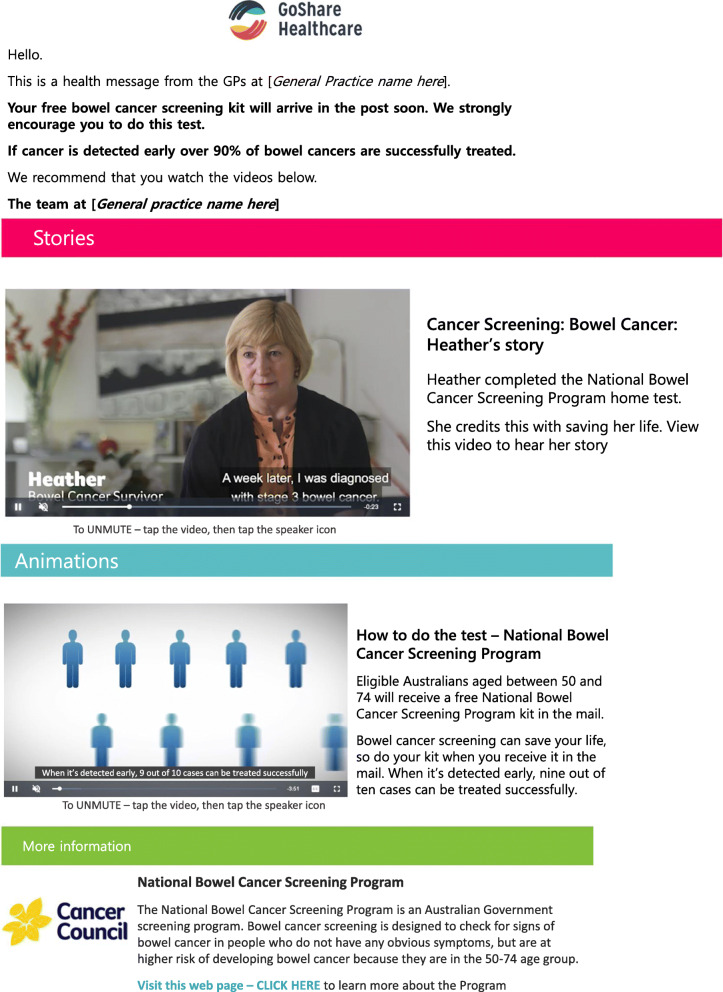
SMARTscreen SMS. (1) A message from the patient’s GP endorsing the NBCSP. (2) Narrative communication video. (3) An animated video describing step-by-step how to do the NBCSP test. (4) Cancer Council Victoria information about bowel cancer screening and the NBCSP

The SMARTscreen SMS is a text message (Fig. 1) which has an embedded hyperlink to a customised webpage with the four evidence-based health information interventions (Fig. 2). The text message is addressed to the patient (using their first name) and from their general practice.

The ‘opt out’ instructions will also in the text message for patients who no longer want to receive SMS communication from the general practice.

The SMARTscreen intervention webpage includes the following (Fig. 2):

1. *A message from their GP endorsing the NBSCP*: The message from the general practice includes the patient’s general practice name and logo. The message encourages the patient to do the bowel cancer screening kit and recommends the patient look at the other interventions in the link.
2. *Narrative communication video*: Directly under the GP message the patient can watch a video of a person describing their positive experience of participating in the NBCSP. The running time for this video is 29 s.
3. *An animated video about how to do the NBSCP test*: This animation guides the patient through how to do the kit and send it back using simple step-by-step instructions. This video was produced by the NBCSP and the running time is 2.47 min [4].
4. *NBCSP information*: A hyperlink to the Cancer Council Victoria website providing information about bowel cancer screening and the NBCSP [28].

The SMARTscreen intervention will be co-designed by Healthily and the Investigator team which is made up of a team of health professionals (GPs, nurses, gastroenterologists), primary care cancer researchers, medical software developers and consumer representatives. The consumer representatives were invited to be investigators as they were directly involved in the development of the video messaging based on their experience of CRC. Pretesting of the initial SMARTscreen SMS will be conducted with the Primary Care Collaborative Cancer Clinical Trials Group (PC4 CAG) including their consumer representative group (PC4 CAG) [29], and GP representatives’ group (GP Circle) [30].

### Criteria for discontinuing or modifying allocated interventions {11b}

General practices will be able to withdraw from the SMARTscreen yrial at any time without providing a reason. Patients may elect to not be contacted by SMS, by replying “STOP” to text message or they may withdraw by contacting their general practice. Patients will not be able to withdraw data as all data will be non-re-identifiable and aggregated.

### Strategies to improve adherence to interventions {11c}

The trial coordinator will be in regular contact with general practice staff delivering the intervention. Support will be provided to deliver the intervention each month and collect the aggregated data. The trial coordinator will be in contact with the control group at two time points throughout the trial to collect aggregated, deidentified data. Training and a comprehensive training manual will be provided to maintain consistency and quality of the intervention delivery and data collection across general practices.

### Relevant concomitant care permitted or prohibited during the trial {11d}

There are no interventions that will be permitted or prohibited during the trial. Usual care will continue to be provided in all participating general practices.

### Provisions for post-trial care {30}

The Healthily Go Share Plus messaging platform, the SMARTscreen SMS and training manual will be available to all participating general practices at the conclusion of the trial if the intervention is found to be efficacious. All participating general practice staff will receive a report for the SMARTscreen trial which includes detailed results of the study.

There is minimal risk to patients who receive the SMS. The SMARTscreen recommendations are within the Australian Colorectal Cancer Guidelines [31] and participants will be due to receive a NBCSP kit within a month of receiving the SMS. A poster in the general practice waiting room will inform patients that the general practice is participating in research and some patients aged between 50 and 60 years old will receive an SMS and if they have any questions to discuss this with the practice manager. All SMSs include the general practice telephone number. General practice staff will be trained to provide accurate information about the trial if patients contact their general practice with questions.

### Outcomes {12}

1. The primary outcome will be the difference in the proportion of eligible patients who completed an FOBT between the intervention and control group for up to 12 months after the SMS was sent as recorded in their EMR.
2. The process measures will be evaluated quantitatively and qualitatively for the intervention group only.

Quantitative process measures collected by age and gender for each general practice will be:

- The number of patients sent a SMS;
- The number of patients sent a SMS and opted out;
- The number of patients sent a SMS and opened the hyperlink to view the GP message;
- The number of patients sent a SMS and opened the hyperlink to view the narrative video and how many times they view the narrative video;
- The number of patients sent a SMS and opened the hyperlink to view the animation of the step-by-step instructions to complete the FOBT and how many times they did this; and
- The number of patients sent a SMS and opened the hyperlink and then click on the link to view the NBCSP webpage information and how many times they did this.

Qualitative process measures will be collected using interviews based on Sekhon’s framework for acceptability [32] and Murray’s Normalisation Process Theory [33] to explore feasibility. The interviews will be used to understand if and how the SMS might work in general practice from the practice staff and the patients’ perspectives. This will also provide important information about barriers and facilitators to embedding the SMS into routine clinical practice (Additional Documents B & C).

### Participant timeline {13}

The participant timeline is shown in Table 1.

**Table 1:**
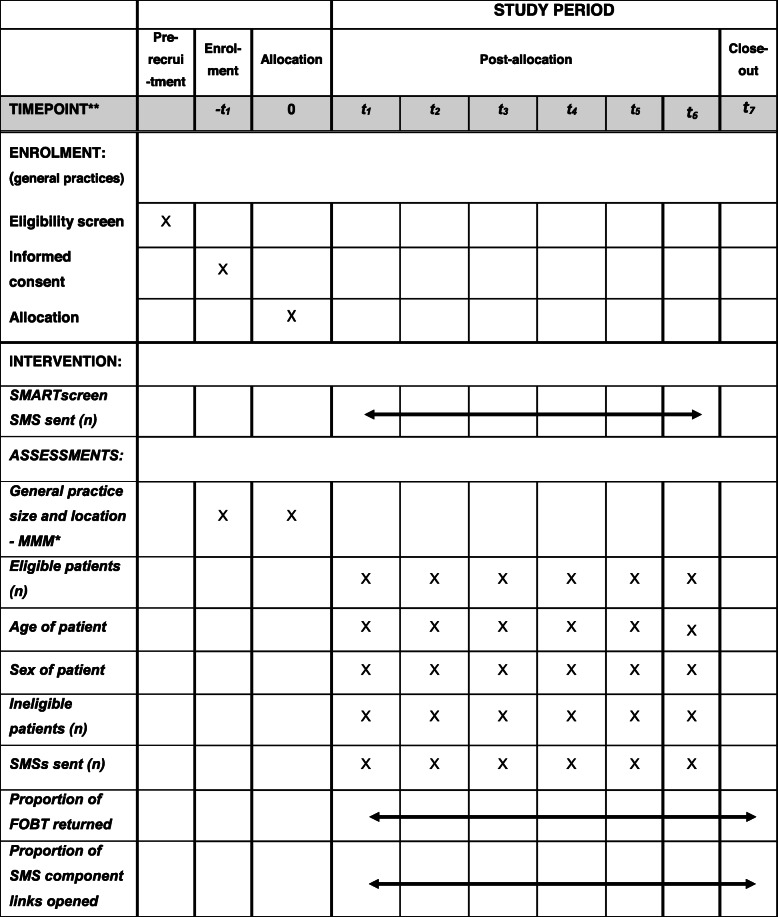
Participant timeline

### Sample size {14}

In total, we require 1400 eligible patients (70 per practice) from 20 general practices to provide 80% power with two-sided 5% significance level to detect a 10% increase in bowel screening participation in the intervention group compared to the control group (50% vs 40%). The estimates are based on average general practice population size, assuming an intra-cluster correlation of 0.008 based on previous studies in general practice [34]. We have not allowed for attrition as the primary outcome data will be collected from the EMR and we anticipate no loss of practices because of the minimal requirement of input from general practice staff.

We aim to recruit up to 20 practice staff and 20 patients (or until data saturation is reached) from the intervention group for the interviews.

### Recruitment {15}

Potentially eligible general practices will be identified through VicRen, the University of Melbourne Department of General Practice research and education network [35]. The trial coordinator will recruit purposively to ensure a diverse sample by rurality and size of general practice is achieved. All participating general practices will be reimbursed $AUD 500 for their involvement in the trial in recognition of the administrative costs involved in participation.

Recruitment of interviewees is described previously.

## Assignment of interventions: allocation

### Sequence generation {16a}

The unit of randomisation (cluster) will be the general practice. Once consented, general practices will be randomly allocated with a 1:1 ratio to either the intervention or control group. Using a computer-generated allocation sequence, stratified by geographical remoteness (two strata defined below) and general practice size (< 1000 patients vs ≥1000 patients), with random permuted block sizes within each stratum. Geographical remoteness will be classified using the Modified Monash Model (MMM) classification [36], as either metropolitan/regional cities (MMM 1-3) or rural town/remote communities (MMM 4&5) General practice size will be determined by the number of active patients aged 50 to 60 years old.

### Concealment mechanism {16b}

To ensure concealment the block sizes will not be disclosed. The statistician will be blinded to the allocation of the general practices using a code of 1 and 2 for the two trial groups in the generated allocation sequence. The key to the code for the trial groups will be retained by the study coordinator. Once the practice has been informed about the allocation, the trial coordinator will record the practice’s code and trial group allocation but will not report the allocation to the statistician.

### Implementation {16c}

A statistician who is blinded to the identity of the participating general practices and not involved in the trial recruitment and data collection will generate the allocation sequence. The statistician will inform the trial coordinator of the randomisation outcome of each general practice who will inform the practice manager of their general practice trial allocation both in writing and verbally.

## Assignment of interventions: Blinding

### Who will be blinded {17a}

General practice staff and patients cannot be blinded to the study group allocation due to the nature of the intervention. The statistician and investigators not involved in the delivery of the intervention will remain blinded to the study group status using a code. The key to the code will be securely stored by the trial coordinator.

### Procedure for unblinding if needed {17b}

The study group status code will only be revealed to the investigators and statistician after all the primary outcome data have been collected and the results presented to the investigators.

## Data collection and management

### Plans for assessment and collection of outcomes {18a}

Demographic data collected at baseline will describe each general practice by size, determined by the number of patients aged between 50 and 60 years old and the location of the general practices as classified using the Modified Monash Model [36]. Data collection will be undertaken at the general practice by the practice manager. Data will be collected from the CAT4 clinical audit tool and using the SMARTscreen filters. These data include the number of eligible patients in each age group (50, 52, 54, 56, 58, 60 years old); their sex; if they have a mobile telephone number recorded and if they have opted out of receiving SMS messages from the general practice. Each general practice staff member participating in the SMARTscreen trial will be trained to apply the filters, use CAT4 and collect these data. Data will be collected monthly in intervention general practices and at two 6-monthly time points in control group general practices. Data collection related to the use of the SMS intervention will be automatically collected by GoShare Plus and is the number of SMS sent; the number of SMS opened and the number of times each of the four intervention components are viewed. All data will be de-identified for each age group (50, 52, 54, 56, 58, 60 years old) and sex (male/female/other) by the general practice.

Qualitative data will be collected from semi-structured interviews. All interviews will be audio-recorded and transcribed verbatim using a University of Melbourne preferred transcribing agency. All transcripts will be anonymized.

### Plans to promote participant retention and complete follow-up {18b}

The trial coordinator will be in regular contact with the general practice staff in the intervention group throughout the 6-month period when they are sending out the SMSs. This will ensure any troubleshooting of problems are managed immediately and also ensure the fidelity of the intervention and data. During this time the trial coordinator will keep records of any deviations from the trial or problems encountered which will be included in the results as part of the process evaluation.

### Data management {19}

All data will be collected by the trial coordinator from the general practices and from Healthily. All data will be de-identified and aggregated. Data management will be done by the trial coordinator and all de-identified data will be stored on University of Melbourne password protected computers with a secure two-factor authentication for extra security.

### Confidentiality {27}

All research data collected will be stored in accordance with the University of Melbourne’s Research Data Management Policy and Research Code of Conduct. Any identifiable data such as consent forms will be stored on the University of Melbourne managed storage infrastructure which are password protected and restricted.

All interview data will be presented after analysis and will not include any identifying information. Participants will be represented by pseudonyms and combined characteristics when using examples so they cannot be identified. All data will be destroyed 5 years after publication according to the University of Melbourne Office of Research Ethics and Integrity Ethics Committee (OREI)

### Plans for collection, laboratory evaluation and storage of biological specimens for genetic or molecular analysis in this trial/future use {33}

Not applicable. No biological specimens were collected.

## Statistical methods

### Statistical methods for primary and secondary outcomes {20a}

Descriptive statistics will be used to summarise the general practice characteristics, patients age and sex for both trial groups. We will also compare the proportion of active patients aged 50 to 60 years old and due to a NBCSP kit that was excluded because they did not have a mobile telephone number recorded in their EMR record, or opted out of receiving SMS messages from their general practice.

Intention-to-treat analysis will be used, where all general practice allocated to the trial group will be included and analysed in the group they were assigned [37]. The primary outcome will be calculated as the proportion of eligible patients who complete the FOBT up to 12 months following the beginning of the trial intervention for both the intervention and control groups. The primary outcome will be compared between the intervention and control groups using logistic regression and generalised linear model with an identity link function and binomial family to estimate the odds ratio and difference in proportions, respectively. Both regression models will use generalised estimating equations with robust standard errors to allow for clustering by general practice and will adjust for the randomisation stratification factors (geographical remoteness using the Modified Monash Model) and general practice size. Both the relative and absolute measures of the estimated intervention effect will be reported with respective 95% confidence intervals, and a *p* value calculated from the logistic regression model. The intra-practice correlation, which quantifies the proportion of the total variation in the outcome attributable to between-cluster variation in the outcome, will also be estimated using one-way analysis of variance and reported with 95% confidence intervals. Statistical analysis will be conducted using Stata statistical software 15 [38]. Process measures will be summarised as counts and proportions of active patients who opened each of the four intervention components of the SMARTscreen intervention SMS at least once, and how many of the components were opened (none, 1, 2, 3 or 4). We will also summarise the number of times the links to the four interventions were accessed.

Qualitative acceptability and feasibility outcomes will be described by themes from the inductive analysis of interviews with general practice staff and patients.

### Interim analyses {21b}

Not applicable; no interim analysis is planned.

### Methods for additional analyses (e.g. subgroup analyses) {20b}

No applicable, no other data will be collected.

### Methods in analysis to handle protocol non-adherence and any statistical methods to handle missing data {20c}

We do not anticipate missing data as the data will extracted from the EMR. We will not perform non-adherence analysis because the primary outcome cannot be linked to the level to identify which patients accessed the SMARTscreen intervention.

### Plans to give access to the full protocol, participant level-data and statistical code {31c}

The data including the statistical code will not be available for public access.

## Oversight and monitoring

### Composition of the coordinating centre and trial steering committee {5d}

The SMARTscreen trial is overseen by a steering committee including JM, AW, JE, MJ, TC, EW, PC, CO, TC, ID, JT, JMG, LG, HK, and GB. The steering committee are responsible for guiding the design and conduct of the trial, development of the intervention, preparation of the protocol and publication of results, budget, and contractual administration, and managing the trial coordinator. The steering committee will be responsible for reviewing progress of the study and if necessary, agreeing on changes to the protocol to facilitate the smooth running of the study.

The trial coordinator will be responsible for running the day-to-day trial activities, completing ethics applications, registering the trial, organising steering committee meetings, recruiting general practices, coordinating the allocation of the randomisation, liaising with the general practice staff and software companies to troubleshoot any problems, providing the funding body quarterly reports, data collection, liaising with the principal investigator and reporting back to the steering committee.

### Composition of the data monitoring committee, its role and reporting structure {21a}

PC, JM, JE, MJ and AW will report to the steering committee as to the data collection and analysis plan.

### Adverse event reporting and harms {22}

Any adverse events and other unintended effects that may arise from the trial intervention will be reported to the University of Melbourne Office of Research Ethics and Integrity Ethics Committee (OREI).

### Frequency and plans for auditing trial conduct {23}

Progress reports will be submitted quarterly to the funding body - The Victorian Cancer Agency (VCA) annually to the University of Melbourne Office of Research Ethics and Integrity (OREI) and regularly to the Australian and New Zealand Clinical Trials Registry (ANZCTR). This will be completed by the trials coordinator (AW) and overseen by the principal investigator (JM).

### Plans for communicating important protocol amendments to relevant parties (e.g. trial participants, ethical committees) {25}

Protocol amendments will be communicated to the steering committee by email and at quarterly meetings. The core team of AW, JM, JE, MJ, EW, TC, and PC will meet fortnightly to discuss any modifications to the trial protocol or trial update on the progress of intervention and data collection. The trial coordinator will communicate with the rest of the Steering Committee to ensure they are all involved in the decision making. The trial coordinator will also update the funding body (VCA), EROI and the ANZCTR with modifications to the protocol or progress of the trial as necessary. All protocol amendments will be reported to the University of Melbourne Office of Research Ethics and Integrity (OREI).

### Dissemination plans {31a}

The final results will be published in scientific peer-reviewed journals and presented at scientific conferences. We will also disseminate our findings through our research and professional networks including the Primary Care Collaborative Cancer Clinical Trials Group (PC4), VicREN, The Department of General Practice University of Melbourne seminar series, the Cancer Council Victoria communication networks, the WVPHN newsletters, Monash University ‘Lens’, The University of Melbourne and research reports will be provided to the general practices involved in the study.

## Discussion

Colorectal cancer screening among 50 to 74 years old is recommended in the Australian Colorectal Cancer Screening Guidelines and increasing the uptake of the NBCSP is a national priority [31]. We are testing whether the SMARTscreen intervention is an efficacious method for increasing uptake of the NBCSP and to explore process measures to understand the barriers and facilitators to implement the SMS into general practice.

We chose to use a multi-faceted intervention to maximise the effect of multiple evidence-based interventions. A recent systematic review and meta-analysis found that combining multiple evidence-based interventions that address known barriers are more effective in increasing CRC screening uptake than using single strategies alone [20]. Qualitative studies highlight a need for accessible information about bowel cancer in a visual format and simplified diagrammatic steps for doing the test along with GP endorsement [39, 40]. The challenge is to determine the selection of, and the ideal format for the delivery of these interventions to people eligible to participate in CRC screening.

SMARTscreen has several strengths. We are using a cluster randomised controlled trial design to investigate the impact of our intervention on bowel cancer screening uptake to minimise the risk of contamination. Secondly, we will have process data about which components of the SMARTscreen intervention are viewed by patients and how often, allowing us to understand the acceptability of our intervention and which components are accessed and viewed. Thirdly, we will obtain richer qualitative data from a range of patients and primary care provider perspectives providing greater insights into the feasibility, acceptability and impact of each intervention component.

## Conclusion

Increasing participation in the NBCSP is critical to maximise the program’s cost-effectiveness and reduce mortality from bowel cancer in Australia. This trial will inform whether an SMS combined with GP endorsement, information and patient narrative can increase uptake of the NBCSP among 50- to 60-year-old patients. It will provide evidence about the feasibility of implementing this approach in general practice. The results of this feasibility trial will also inform decisions about the need for and design of a larger, multi-state trial of the SMARTscreen intervention to determine its effectiveness and cost-effectiveness and important implementation methods. If successful, we believe the intervention is scalable and easily transferable to other types of cancer screening.

## Trial status

We commenced SMARTscreen in 2020 during which the NBCSP continued in Australia despite the COVID-19 pandemic. By October 2020, a total of 21 general practices were recruited to SMARTscreen with an 84% recruitment success. Although our original sample size was 20 general practices, the SMARTscreen trial steering committee agreed to recruit all 21 general practices who expressed interest, to allow for potential attrition. The intervention period commenced in January 2021 and will be completed by the end of July 2021. Data collection will continue until the end of December 2021 and trial results are due in 2022.

## Data Availability

The trial data set will be available to the trial coordinator and the statistician. These data including the statistical code will not be available for public access.

## Abbreviations

ANZCTR: Australia New Zealand Clinical Trial Registry
CAT4: Clinical Audit Tool 4
CRC: Colorectal cancer
EMR: Electronic Medical Record
EMS: Electronic meeting service
FOBT: Immunochemical Faecal Occult Blood Test
GP: General practitioner
NBCSP: National Bowel Cancer Screening Program
RCT: Randomised controlled trial
SMS: Short messaging service
VCA: Victorian Cancer Agency
WVPHN: Western Victoria Primary Health Network
